# Oxygen-Sufficient Nanoplatform for Chemo-Sonodynamic Therapy of Hypoxic Tumors

**DOI:** 10.3389/fchem.2020.00358

**Published:** 2020-04-28

**Authors:** Biying Huang, Sijie Chen, Wenjing Pei, Yan Xu, Zichao Jiang, Chengcheng Niu, Long Wang

**Affiliations:** ^1^Department of Ultrasound Diagnosis, The Second Xiangya Hospital, Central South University, Changsha, China; ^2^Department of Orthopedics, Xiangya Hospital, Central South University, Changsha, China

**Keywords:** chemo-sonodynamic therapy, perfluorocarbon, tumor hypoxia microenvironment, reactive oxygen species, oxygen sufficient nanoplatform

## Abstract

Modulation of hypoxia is an essential factor for enhancing the effects of antitumor therapies, especially sonodynamic therapy and chemotherapy. To improve the efficacy of combination therapy by reversing the hypoxic tumor microenvironment, we developed shell-core structured PPID-NPs, which were designed with a polymer shell onto the sonosensitizer and a chemotherapeutic drug were loaded and a perfluorocarbon core loaded with oxygen. The perfluorocarbon core provides sufficient oxygen not only for causing the sonosensitizer to produce more singlet oxygen to induce cell apoptosis but also for reducing drug resistance to enhance therapeutic efficacy. Furthermore, the release of chemotherapeutic drugs at the tumor site can be controlled. Thus, PPID-NPs can efficiently inhibit the growth of breast cancer by synergistic therapy under ultrasound exposure. We believe that our oxygen-sufficient nanoplatform could be an ideal therapeutic system for hypoxic tumors.

## Introduction

Tumor hypoxia results from deteriorating diffusion geometry, structural abnormalities of tumor vessels, and disturbed microcirculation (Hockel and Vaupel, 2001). It is widely accepted that hypoxia can promote tumor propagation and malignant progression (Unruh et al., 2003; Vaupel and Mayer, 2007). Moreover, hypoxia can induce resistance to therapy, such as chemotherapy (Unruh et al., 2003; Cheng et al., 2015; Chen et al., 2017) and sonodynamic therapy. In sonodynamic therapy, which was developed from photodynamic therapy, a sonosensitizer can translate energy absorbed from ultrasound (US) into oxygen to produce reactive oxygen species (ROS). The insufficient oxygen supply in tumors greatly hinders the efficacy of sonodynamic therapy. Moreover, during the SDT process, a large amount of oxygen is consumed, which worsens the hypoxic tumor microenvironment. Therefore, it is essential to develop ways of overcoming this obstacle. Hyperbaric oxygen (HBO) therapy was first exploited to modulate tumor hypoxia (Al-Waili et al., 2005); however, HBO causes lung injury and neurotoxicity, significantly limiting its clinical application (Saugstad, 2012). To date, nanomaterials have been synthesized to deliver or produce oxygen, including calcium peroxide (CaO_2_), manganese dioxide (MnO_2_), hemoglobin, and hydrogen peroxide (H_2_O_2_)- or catalase-loaded nanoparticles (Chen et al., 2016; Liu et al., 2017; Gu et al., 2018; Jia et al., 2018; Xu et al., 2018; Yang et al., 2018; Zhu et al., 2018; Sun et al., 2019), which hold promise for improving therapeutic outcomes. Among these methods, some studies have taken advantage of endogenous H_2_O_2_ in the tumor, which reacts with either catalase or MnO_2_ nanoparticles to produce oxygen. However, these methods are restricted by the amount of H_2_O_2_ available in tumors (Song et al., 2016), the poor biocompatibility of Mn^2+^ itself and their insufficient oxygen delivery efficiency. There are some other alternatives proposed as “blood substitutes,” such as hemoglobin-based oxygen carriers and perfluorocarbon-based oxygen carriers. Hemoglobin-based oxygen carriers are able to load oxygen under a high oxygen partial pressure (pO_2_) in the lungs and release it under the lower pO_2_ in the tissue (Luo et al., 2018). However, without undergoing a reduction, free Hb in circulation rapidly becomes the met form and releases toxic free heme, causing kidney tubule damage and even renal failure (Buehler et al., 2007, 2010), vasoconstriction and systemic hypertension (Bialas et al., 2019). Therefore, more efforts should be made to search for effective and highly biosafe oxygen carriers. Perfluorocarbon (PFC) compounds are chemically and biologically inert synthetic materials with high oxygen solubility. Compared to Hb, PFCs can dissolve large amounts of oxygen without saturation instead of reversibly binding oxygen. In particular, PFCs are stable under processing, storage, and usage conditions and are not prone to oxidation. Moreover, with the augmented fraction of inspired oxygen (FiO_2_), PFC can load more oxygen in circulation and rapidly diffuse to tissues via an O_2_ gradient (Cabrales et al., 2004; Bialas et al., 2019). Surprisingly, in addition to the characteristics mentioned above, perfluorotributylamine (PFTBA), one type of PFC, has a platelet inhibition effect that can enhance RBC infiltration at tumor sites to promote oxygen delivery (Zhou et al., 2018). Therefore, PFTBA holds great potential in delivering oxygen to tumors.

**Figure 1:**
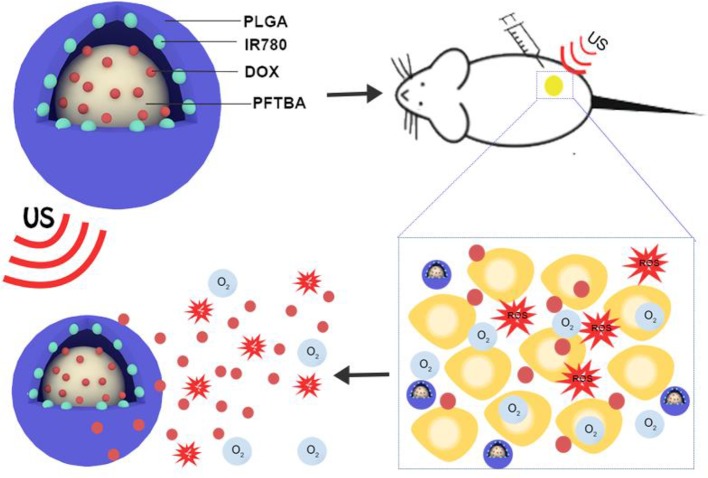
Schematic illustration of the structure of PPID-NPs, ROS production and drug release process under ultrasound exposure.

To date, many studies on tumor therapy have focused on designing combination therapies, especially SDT and chemotherapy (Qian et al., 2016; Lin et al., 2019). In the combination therapy between SDT and chemotherapy, ROS are generated under US irradiation, which can exert killing cell ability (Bai et al., 2012; Pan et al., 2018). With the help of US exposure, the release of a chemotherapeutic drug at the tumor would be promoted, and the chemo-drug resistance of cancer cells would be reversed (Wu et al., 2019). However, although *in vivo* results have shown a better therapeutic effect comparable to that of single therapy, hypoxia is still an obstacle that weakens the therapeutic efficacy (Shen et al., 2015; Wu et al., 2019). In this study, encouraged by the outstanding characteristic of PFTBA, we built an oxygen nanoplatform (PPID-NPs) to augment the efficacy of sonodynamic-chemotherapy against breast cancer. In this nanoplatform, we designed core-shell nanoparticles with a polymer shell and a PFTBA core, in which the sonosensitizer IR780, doxorubicin hydrochloride (DOX) and oxygen were loaded (Figure 1). The shell of these NPs was composed of an FDA-approved polymer, poly(lactic-co-glycolic) acid (PLGA), with high biodegradability and excellent biocompatibility. The core of these NPs was composed of PFTBA as the oxygen carrier. DOX, as a broad-spectrum antitumor drug, was encapsulated in these NPs to enhance the efficacy of sonodynamic-chemotherapy. The sonosensitizer IR780, as a prototypical near-infrared (NIR) heptamethine cyanine agent, has been greatly explored for anticancer therapy, including sonodynamic and photodynamic therapy (Thomas et al., 2017; Zhang et al., 2019). However, its poor solubility in biological fluids, fast clearance, and acute toxicity (at high doses) hinder the further use of IR780. To address these critical limitations, IR780 can be loaded into our nanoplatform to achieve high solubility, low toxicity, and long residence time (Alves et al., 2018). In this work, we successfully prepared the oxygen-sufficient nanoplatform PPID-NPs, and *in vitro* and *in vivo* experiments verified their wonderful oxygen-loading capacity and excellent anticancer performance. Thus, PPID-NPs can be applied as useful agents for enhancing the efficacy of chemo-sonodynamic therapy against hypoxic tumors.

## Materials and Methods

### Materials

IR-780 iodide, PLGA, and polyvinyl alcohol (PVA) were purchased from Sigma-Aldrich (USA). Liquid PFTBA was obtained from BioRike (China). DOX was purchased from Solarbio Co. Ltd. (China). A reactive oxygen species assay kit DCFH-DA was purchased from Beyotime Biotechnology (China), and the singlet oxygen sensor green (SOSG) probe was provided by Thermo Fisher (USA). Other reagents were of analytical purity and were used without further purification.

### Preparation of PPID-NPs

Nanoparticles were prepared using a single emulsion evaporation method based on our group's previous study (Wang et al., 2018). Briefly, 25 mg of PLGA was completely dissolved in chloroform, and then 1 mg of IR780, 1 mg DOX of (dissolved in 100 μL of deionized water) and 100 μL of PFTBA were added to the PLGA solution. Finally, 8 mL of 4% w/v PVA solution was added to the PLGA solution and emulsified for 2 min with an ultrasonic processor in an ice bath. The resulting emulsion was mixed in 10 mL of deionized water and stirred for 3 h. Next, the resulting NPs were washed with deionized water (10 000 rpm, 20 min) until the supernatant became colorless. When the precipitate was resuspended with PBS, PPID-NPs were pre-saturated with a medical oxygen cylinder for 15 min in an ice bath and stored at 4°C. We termed the NPs as PPID-NPs. All procedures were performed in the dark. The same procedure was used to prepare PPI-NPs without DOX, PI-NPs without DOX and PFTBA, and PLGA/PFTBA NPs without DOX and IR780. These NPs were used as controls.

### Characterizations

The morphology of the PPID-NPs was observed by transmission electron microscopy (TEM, Jem-1400 plus). The size distribution, polydispersity, and zeta potential were analyzed by a Malvern size analyzer (ZEN3600, Malvern Instruments, US). Stability experiments of the NPs were performed in 1× PBS or in 10% fetal bovine serum (FBS) with a dynamic laser scattering (DLS) instrument over 7 days. The presence of IR780 in the NPs was verified on a UV–vis–NIR spectrophotometer (UV-2450, Shimadzu, Japan). The presence of DOX in the NPs was identified using a fluorescence spectrophotometer (F-4600 FL, Hitachi, Japan).

### Determination of IR780/DOX Entrapment Efficiency and Loading Efficiency

A 10 mg/mL nanoparticle solution was subjected to lyophilization. After 24 h, they were weighed, dissolved in DMSO, and diluted to a suitable concentration, and the absorbance values at the maximum absorption peak positions of IR780 and DOX were measured by ultraviolet-visible spectrophotometry. The concentration was calculated using the corresponding standard curve.

$$\begin{array}{l} \text{Encapsulation efficiency}\left(\text{\%}\right)=W_{E}/W_{O}\times 100\left(\%\right)\\ \text{Drug loading efficiency}\left(\text{\%}\right)=W_{E}/W_{N}\times \;100\left(\%\right) \end{array}$$

*W*_*E*_: weight of IR780 (DOX) in nanoparticles after lyophilization

*W*_*O*_: original input of IR780 (DOX)

*W*_*N*_: weight of the nanoparticles after lyophilization.

### DOX Release From PPID-NPs With US Irradiation

The drug release experiment was divided into two groups: (1) PPID-NPs without US irradiation and (2) PPID-NPs with US irradiation. First, PPID-NPs (25 mg) were reconstituted in 1 ml of PBS (PH = 7.4) and loaded into a dialysis bag (M_w_ = 8,000 Da). Group 2 was subjected to US irradiation (2.0 W/cm^2^) for 5 min. The two groups were placed on a shaker whose speed was 100 rpm. At the desired time points, 1 mL of dialysate was removed, and then fresh buffer was added back to the reservoir. Each DOX concentration in the dialysate was obtained by a fluorescence spectrometer, and the accumulative amount of DOX released was calculated according to the standard curve. Each group was tested in triplicate, and the data are given as the mean ± SD.

### Measurement of ROS in the Cell-Free System

The production of ^1^O_2_ was measured using SOSG as a fluorescent probe. This experiment was divided into three groups: (1) PPI-NPs, (2) PI-NPs (10 mg/mL), and (3) PBS. For each group, 100 μL of solution was added to 1 ml of PBS. Then, 1 μL of SOSG (2.5 mM) in methanol was mixed in a quartz cuvette. The US irradiation time (1 MHz, 1 W/cm^2^, 40% duty cycle) using a US transducer (WED-100, WELLD Medical Electronics, China) was 0, 30, 60, 90, 120, or 150 s. The fluorescence spectra of SOSG were acquired on a fluorescence spectrometer with an excitation wavelength of 504 nm.

$$\begin{array}{l} \text{Relative}{\;}^{1}{\text{O}}_{2}\text{ production efficiency}:\text{F}/{\text{F}}_{0} \end{array}$$

F: fluorescence intensity of the three groups under US irradiation

F_0_: fluorescence intensity of the three groups before US irradiation.

### Oxygen Storage and Release of PPID-NPs

The oxygen delivery experiment was divided into three groups: (1) PPID-NPs, (2) PID-NPs, (3) Water. First, 1 mL of each group were oxygenated by a medical oxygen cylinder for 5 min in a gas bottle. Second, 4 mL of degassed water was loaded into the glass bottle with a rubber topper, and a 50 ml syringe was used to exhaust the gas in the bottle to simulate a hypoxic environment. Then, the oxygenated solution was rapidly transferred into the bottle. The real-time oxygen concentration in the bottle was measured by a portable oxygen analyzer (AMT08 DO Meter). The oxygen concentration was recorded every 10 s.

### Cell Culture

For cell culture under hypoxic and normoxic conditions, 4T1 breast cancer cells and MCF-7 cells were cultured in 1,640 medium in an atmosphere of 21% O_2_ and 5% CO_2_ at 37°C to mimic a normoxic environment. For comparison, an atmosphere of <5% O_2_ at 37°C was used to mimic the hypoxic tumor microenvironment.

### Cellular-Level ROS Generation

A cellular level ROS assay kit, DCFH-DA, was used to detect intracellular ROS production. 4T1 cells (1 × 10^5^ cells per well) were seeded in six-well plates and randomly divided into five groups: (1) PBS, (2) US-only, (3) PPI-NPs, (4) PI-NPs + US, and (5) PPI-NPs + US. After 24 h of incubation, DCFH-DA (10 μM) was added to each well. Then, the corresponding treatments were administered to each group. Finally, ROS production was determined by fluorescence microscopy after three washes with PBS.

### Cellular Apoptosis Experiment

4T1 cells (1 × 10^5^ cells in each well) and MCF-7 cells were seeded in 6-well plates, cultured overnight, and then divided into five groups: (1) PBS, (2) US-only (1.0 W/cm^2^, 2 min), (3) PPI-NPs, (4) DOX, (5) PPID-NPs + US (1.0 W/cm^2^, 2 min). The volume of all NP solutions was 150 μL (10 mg/mL), and the content of free DOX was equal to that in the NPs (13.2 μg). Then, all 4T1 cells and MCF-7 cells were washed twice with PBS, stained with propidium iodide (PI) and DAPI, and imaged by fluorescence microscopy.

### *In vitro* Biocompatibility of PPID-NPs

The vitro biocompatibility of PPID-NPs was tested on 4T1 and MCF-7 cells using a standard CCK-8 assay. Cells were seeded in two of 96-well plates with a density of 7 × 10^3^ cells per milliliter and cultured in normoxic and hypoxic environments overnight. The hypoxic environment was created by an AnaeroPack (Mitsubishi Gas Chemical Co, Inc.) and a 2.5 L sealable culture tank; when the color of the oxygen indicator changed from purple to pink, a hypoxic environment was successfully formed, and PPID-NPs in cell culture medium at different concentrations (0, 75, 150, 300, 600, or 800 μg/mL) were added. After 24 h of incubation, the cell viability was measured by a cell counting kit-8 (CCK-8).

### *In vitro* Antitumor Study

4T1 cells and MCF-7 cells were seeded into two 96-well plates (1 × 10^4^ cells) and cultured overnight under normoxic or hypoxic conditions. The cells were randomly divided into six groups: (1) PBS, (2) US-only, (3) DOX, (4) PI-NPs +US, (5) PPI-NPs + US, and (6) PPID-NPs +US. Then, the cells were cultured with NPs (300 μg/mL) in 1,640 medium for 4 h. After discarding the above culture medium, the cells were washed three times with PBS, and complete medium was added to each well. Then, groups (2), (4), (5), and (6) were exposed to US (1 MHz, 1 W/cm^2^) for 2 min. To carry out US irradiation, the space between the probe and plate was full of an ultrasonic coupling agent. After 24 h of incubation, the cell viability was determined using a CCK-8 cytotoxicity assay kit.

### *In vivo* Tumor Hypoxia Environment After Different Treatments

BALB/c mice (5 weeks, female) were obtained from the Laboratory Animal Center of Central South University (China) and maintained in accordance with the guidelines of the Department of Laboratory Animals, Central South University, China. Breast tumor-bearing mice were established by the subcutaneous injection of 4T1 cells (2 × 10^6^) into the right flank, and the tumor volume was calculated according to formula 1/2 a × b × c (a, tumor long diameter; b, tumor short diameter; c, tumor height). When the tumor volume reached 100 mm^3^, the breast tumor-bearing mice (*n* = 3) were divided into three groups: (1) saline, (2) PI-NPs, and (3) PPI-NPs. After the NPs were intratumorally injected, all mice were sacrificed, and tumor tissues were immunohistochemically stained with an antibody against hypoxia-inducible factor-1α (HIF-1α) after 24 h.

### Combination Therapy Between SDT and Chemotherapy *in vivo*

Breast tumor-bearing mice were randomly divided into 5 groups (*n* = 5) when the tumor volumes reached 60 mm^3^: (1) saline, (2) US-only, (3) DOX, (4) PPI-NPs + US, (5) PPID-NPs + US; for each group, 75 μL of the corresponding treatment solution was intratumorally injected into the mice every 2 days. All NPs were at a concentration of 25 mg/mL, with a DOX concentration of 0.22 mg/mL. The tumors of the mice in groups (3), (4), and (5) were exposed to US (2 W/cm^2^, 5 min) after injection. Body weight and tumor volume were recorded every other day for 14 days. One day later, the tumors of the mice were harvested, and hematoxylin and eosin (H&E) staining was performed. A terminal deoxynucleotidyl transferase-mediated dUTP nick-end labeling kit (TUNEL, Promega, Madison, WI) was used to observe cell apoptosis. Tumor cell proliferation was analyzed by tumor immunohistochemical staining with Ki-67 antibody.

### Pharmacokinetics and Biodistribution

Tumor-bearing BALB/c mice were injected with 200 μL of PPID-NPs and PI-NPs (the concentration of IR780 was 2.0 mg/kg) through the tail vein. For biodistribution study, submandibular vein blood of the mice was collected at different time points (0, 4, 12, 24, 48, and 72 h) for UV-Vis-NIR spectrophotometry analysis. After injection of NPs 24 h, a portion of the major organs and the tumors of the mice were collected, lysed in DMSO and homogenized to test the IR780 contents with a UV-Vis-NIR spectrophotometer. The NPs content was calculated from the IR780 content in the blood or organs from a standard curve.

## Results and Discussion

### Characterization

**Figure 2:**
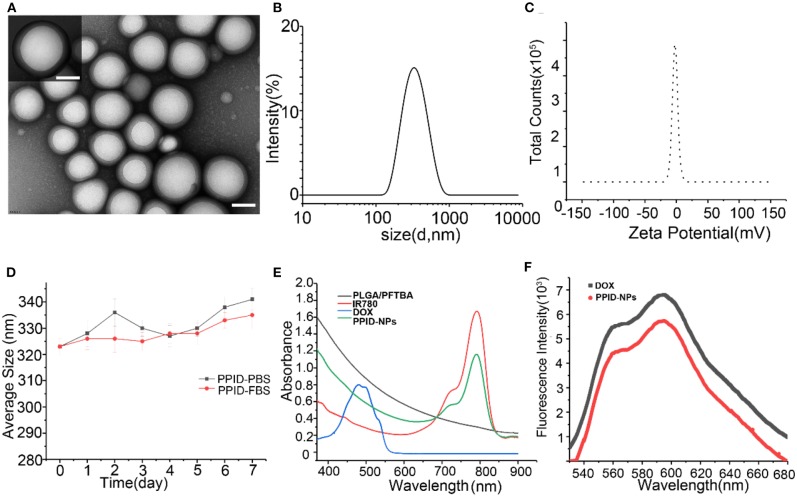
**(A)** TEM images of PPID-NPs (scale bar, 100 nm). **(B,C)** Size and zeta intensity distributions of PPID-NPs. **(D)** Average size of PPID-NPs in 1× PBS or 10%FBS for 7 days. **(E)** UV-vis-NIR absorption spectra of free IR780, DOX, PLGA/PFTBA NPs, and PPID-NPs. **(F)** Fluorescence spectra of PPID-NPs and DOX.

PPID-NPs were prepared by a single emulsion evaporation method. The TEM image in Figure 2A showed that the NPs were spherical. The average size from DLS analysis was 320 nm, and the surface charge was −2.0 mV (Figures 2B,C). The stability of the NPs in PBS and FBS solution were monitored by dynamic light scattering (Figure 2D). The average hydrodynamic diameter of the NPs in PBS solution increased from 323 to 340 nm in a week, accompanied by a polydispersity index (PDI) increase from 0.033 to 0.23. By contrast, the NPs in the FBS solution showed a slight change in hydrodynamic diameter, which increased by approximately 10 nm, and the change in PDI was negligible. This result implied that the PPID-NPs were so stable that they could be studied *in vivo*. From the UV–vis–NIR absorption spectra of PPID-NPs, the PLGA/PFTBA nanoparticles show no absorption intensity in the range of 400–900 nm, free IR780 solution has an absorption peak at 780-795 nm, and DOX has an absorption peak at approximately 480 nm (Figure 2E). The spectrum of the PPID-NPs showed an absorption peak at 780-790 nm, indicating the successful loading of IR780. The fluorescence spectrum of the PPID-NPs showed the same peak of DOX (Ex: 480 nm), which confirmed that DOX was successfully loaded in the NPs (Figure 2F). At a concentration of 25 mg/mL, the IR780 encapsulation efficiency was 54.08%, the drug loading efficiency was 2.0%, the DOX encapsulation efficiency was 22.22%, and the drug loading efficiency was 1.0%.

### DOX Release From PPID-NPs Under US Exposure

**Figure 3:**
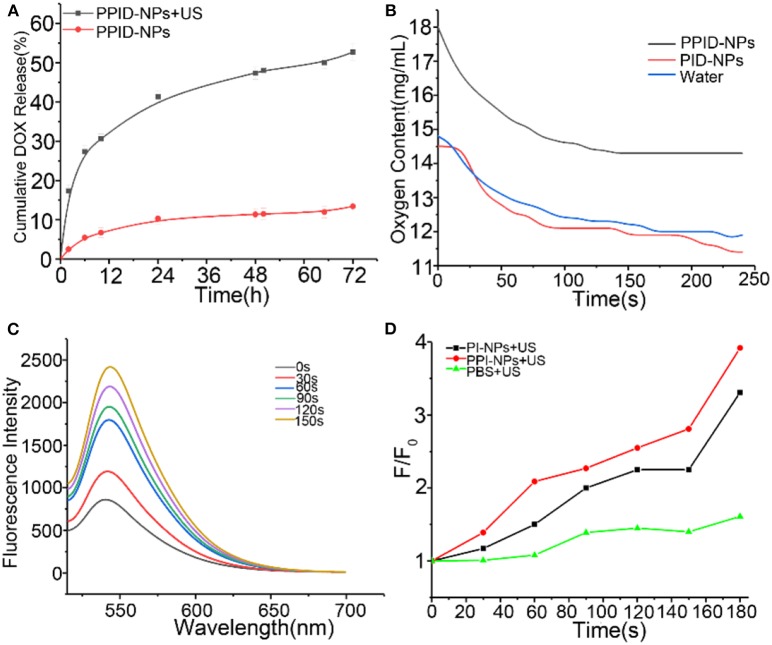
**(A)** After 72 h, DOX release from PPID-NPs with or without US irradiation (1 MHz, 1.0 W/cm^2^). **(B)** Oxygen content of water and PPID-NPs. **(C)** Time-dependent ^1^O_2_ generation of PPI-NPs as irradiated by US (1 MHz, 1.0 W/cm^2^). The concentration of IR780 was 21.6 μg/mL. **(D)** The time-dependent fluorescence increment of SOSG under ultrasound irradiation.

To investigate the DOX release profile from the PPID-NPs, we explored whether US irradiation promoted the release of DOX from the NPs. Compared with PPID-NPs alone (Figure 3A), PPID-NPs with US irradiation exhibited considerably higher DOX release, and the release rate of DOX approached 53% within 72 h. However, for the NP-alone group, the drug release rate was only 12%, revealing that the DOX release rate was quite slow. Furthermore, when the NPs were combined with US irradiation, the DOX release from the NPs was sharply accelerated in 12 h. Hence, US irradiation could greatly enhance the DOX release rate from the NPs.

### Oxygen Delivery Ability of PPID-NPs

The oxygen loading and release profiles of PPID-NPs were analyzed under hypoxic conditions. After oxygen loading, the oxygen concentration in the PPID-NPs group was 18.0 mg/mL, while the oxygen concentration in the degassed water group was only 14.8 mg/mL and PID-NPs only 14.5 mg/mL. In the following 240 s, the oxygen concentration in the PPID-NPs group was obviously higher than that in the degassed water group and PID-NPs group at every time point (Figure 3B). These results verified the good oxygen-loading capacity and sustained oxygen release property of PPID-NPs in a hypoxic environment. Thus, PPID-NPs hold high promise for alleviating hypoxic conditions in tumors and enhancing oxygen-dependent SDT efficacy.

### Singlet Oxygen Production Experiments

The ^1^O_2_ generation by PPI-NPs under US irradiation was measured using SOSG as a probe. At a final IR780 concentration of 21.6 μg/mL, the fluorescence intensity of SOSG at 540 nm increased drastically with prolonged irradiation duration (1.0 W/cm^2^), indicating the excellent ^1^O_2_ generation ability of PPI-NPs (Figure 3C) and further SDT potential against cancer. As shown in Figure 3D, for the PI-NP group (not containing PFTBA) under US irradiation, the fluorescence intensity of SOSG was increased by 320%. Surprisingly, for the PPI-NP group (containing PFTBA) under the same conditions, the fluorescence intensity of SOSG was increased as high as 400%. Therefore, more ^1^O_2_ generation is produced, which can be attributed to the extra oxygen provided by PFTBA in the SDT process. The outstanding oxygen-loading capacity of PFTBA in the NPs conferred enhanced SDT efficiency in the following study.

### *In vitro* Biocompatibility of PPID-NPs

**Figure 4:**
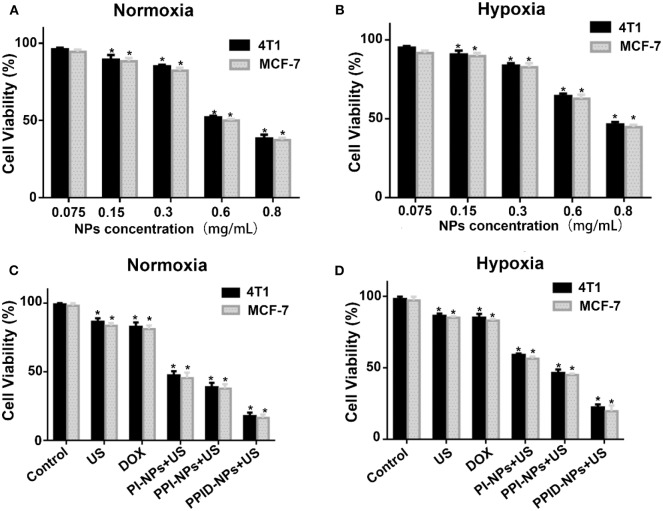
**(A,B)** Cell viability of 4T1 and MCF-7 cells incubated with different concentrations of PPID-NPs under normoxia environment **(A)** and hypoxia environment **(B)** (*p < 0.05, compared with the 0.075 mg/mL nanoparticles concentration group). **(C,D)** Cell viability of 4T1 and MCF-7 cells under different treatments in normoxia environment **(C)** and hypoxia environment **(D)** (*p < 0.05, compared with the control group).

The viabilities of 4T1 cells and MCF-7 cells in normoxic and hypoxic environments were measured via CCK-8 assay. As shown in Figures 4A,B, there was no obvious decrease in cell viability when the NPs concentration was 75 μg/mL under both normoxic and hypoxic conditions, PPID-NPs showed the little toxicity. As the NPs concentration increased, the 4T1 cell viabilities under normoxic conditions gradually decreased to 90.0, 74.2, 62.3, and 43.2%, and the cell viabilities under hypoxic conditions gradually decreased to 87.7, 77.7, 48.7, and 32.3% due to the increasing amount of DOX in the NPs, and MCF-7 cells showed the similar result. To explore the SDT efficiency, we used 300 μg/mL PPID-NPs as an optimum concentration for our subsequent cell experiments.

### *In vitro* Antitumor Activity

The antitumor effect was assessed via a CCK-8 assay under normoxic and hypoxic conditions. As shown in Figure 4C, in the normoxic environment, the 4T1 cell viabilities of the DOX group and US-only group were 82.2 and 86.1%. For MCF-7cell, the cell viabilities of both groups were 83%, which means that both DOX and US exposure had slight toxicity to cells. In the PI-NP or PPI-NP group combined with US exposure, the cell viabilities further decreased to 47.5 and 38.2%, respectively. Due to the SDT effect of the sonosensitizer IR780 in both NPs, ROS are generated and directly kill cells. Compared with the PI-NP + US group, the PPI-NP + US group obviously induced more cell apoptosis, which verified that the PFTBA in the PPI-NPs could load more oxygen into the NPs and thus produce more ^1^O_2_ under US exposure, resulting in much more cell apoptosis. Furthermore, with the help of DOX, the PPID-NP +US group induced apoptosis in a large majority of cells because of the high efficiency of the combinatorial therapy. As shown in Figure 4D, the cell viabilities of the DOX group and US-only group were 85.1 and 83.3%, respectively, in the hypoxic environment. Combined with US exposure, the cell viabilities of the PI-NP group, PPI-NP group and PPID-NP group were 59.5, 46.7, and 21.8%, respectively. These results showed that the cell viabilities determined with the CCK-8 assay in a hypoxic environment were almost consistent with those in a normoxic environment. And the similar result from MCF-7cell was further proved that. Therefore, the oxygen-rich nanoplatform PPID-NPs with US can achieve desirable cytotoxicity in *in vitro* experiments.

### Intracellular ROS Generation

**Figure 5:**
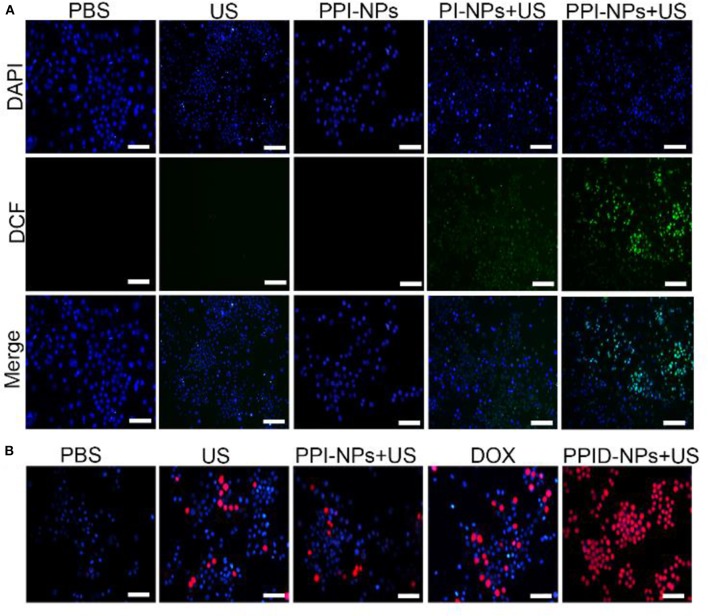
**(A)** Fluorescence microscope images of DCFH-DA stained 4T1 cells subjected to various treatments (scale bar, 100 μm). **(B)** Fluorescence microscope images of DAPI and PI costained 4T1 cells after various treatments (scale bar, 100 μm).

The intracellular sonodynamic activity of PPID-NPs was examined using DCFH-DA as a probe for intracellular oxidative stress. To illustrate the ability of the NPs to produce ^1^O_2_(one type of ROS), we used PPI-NPs without DOX in the following experiments. Cells were randomly divided into five groups: (1) PBS, (2) US-only, (3) PPI-NPs, (4) PI-NPs + US, and (5) PPI-NPs + US. As shown in Figure 5A, 4T1 cells treated with PPI-NPs alone or US irradiation had intracellular green fluorescence as low as that of the blank control. In contrast, green fluorescence was observed in groups 4 and 5, which illustrated that both the sonosensitizer and US exposure were necessary for ^1^O_2_ production. Compared with group 4, group 5 exhibited obviously stronger green fluorescence intensity, which suggested that the PPI-NPs containing PFTBA could carry more oxygen and produce more ^1^O_2_ under US exposure than could PI-NPs without PFTBA loading. Therefore, with the addition of PFTBA, oxygen-loaded NPs had significantly enhanced sonodynamic activity due to the sufficient oxygen supply.

### Cellular Apoptosis Experiment

To explore the potent anticancer activity of the PPID-NPs, a live/dead staining assay was carried out. As shown in Figure 5B, all cells were stained with DAPI, and dead cells were stained with PI. There were a few dead cells (red fluorescence) in the US-only group, suggesting that US irradiation alone could not lead to cell death. When exposed to DOX, some dead cells were observed. Compared with the other groups, the PPID-NP +US group exhibited mainly cells that were destroyed by the combination effect of chemo-sonodynamic activity. This result further confirmed that sonodynamic-chemotherapy has better anticancer efficacy than each single therapy by itself.

### *In vivo* Tumor Hypoxia Environment After Different Treatments

**Figure 6:**
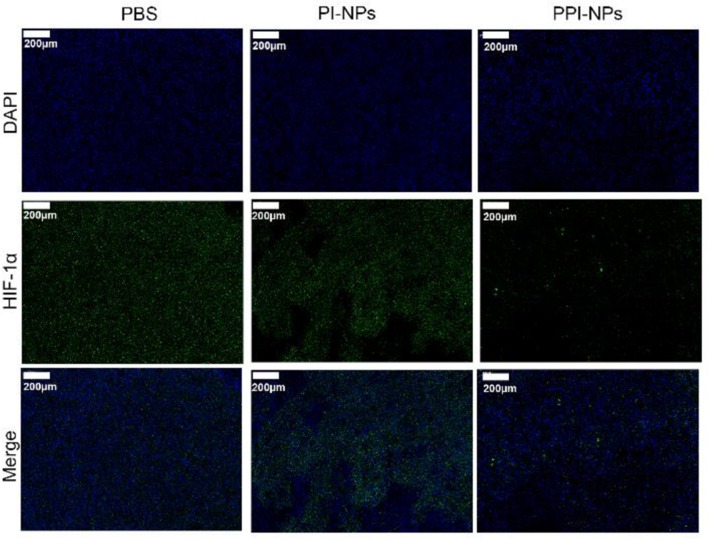
Representative immunofluorescence images of tumor slices stained by the HIF-1α under different treatments (scale bar, 200 μm).

Hypoxia-inducible factors (HIFs) are transcription factors that are expressed in the hypoxic tumor microenvironment (Ajith, 2018). PFTBA was used to store and deliver oxygen to overcome hypoxia in the tumor site. To verify the anti-hypoxic efficacy of the NPs *in vivo*, a HIF-1α probe was applied to evaluate the hypoxia status in the tumor tissues after different treatments: (1) saline, (2) PI-NPs without PFTBA, and (3) PPI-NPs containing PFTBA. As shown in Figure 6, DAPI stained the cellular nuclei blue, and the HIF-1α probe stained the hypoxic cells green. The tumor tissue treated with PBS and PI-NPs displayed strong green fluorescence with high expression of HIF-1α, which indicated hypoxic tumor conditions. Comparatively, for the group treated with PPI-NPs, much weaker and less green immunofluorescence corresponding to HIF-1α expression was observed, demonstrating that tumor hypoxia was significantly ameliorated by PPI-NPs.

### *In vivo* Antitumor Effect

**Figure 7:**
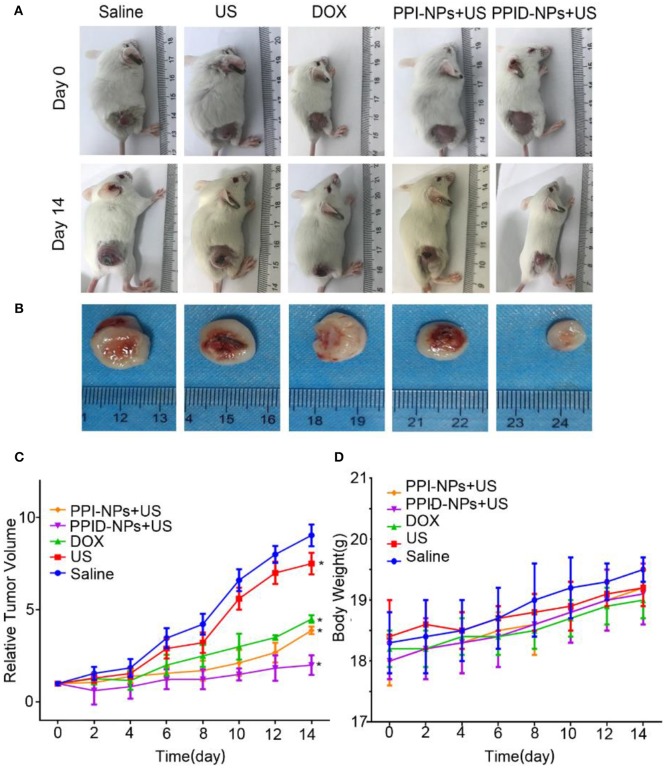
**(A)** The digital photos of representative mice before and after different treatments. **(B)** The digital photos of tumor from mice after treatments at day 14. **(C)** Tumor growth curves of different groups of mice (*n* = 5) after various treatments (values are the mean ± SD, *n* = 5, *p < 0.05, compared with saline, Dunnett's multiple comparisons test). **(D)** The weight growth curves of different groups of mice (*n* = 5) after various treatments.

Encouraged by the excellent *in vitro* performance of the PPID-NPs, then the *in vivo* performance on 4T1 tumor-bearing mice were investigated. The tumor-bearing mice were divided into 5 groups: (1) saline, (2) US irradiation, (3) DOX, (4) PPI-NPs + US, (5) PPID-NPs + US. Digital photographs of tumor-bearing mice at day 0 and day 14 are shown in Figure 7A. The *ex vivo* tumors at day 14 are shown in Figure 7B. After 14 days, compared to the original tumor volume, the tumor volumes increased 9.0-fold for the control group and 8.5-fold for the US group, indicating that US irradiation had no significant antitumor effect (Figure 7C). Compared with the original tumor volume on day 0, the tumor volume on day 14 was increased 4.2-fold for the DOX-only group and 4.0-fold for the PI-NP + US group, which suggested that both DOX and PPI-NPs + US have an obvious antitumor effect *in vivo*. When the mice were treated with PPID-NPs and US irradiation, compared to the original tumor volume, the tumor volume slowly increased 2.0-fold. The *ex vivo* tumors also showed that the tumor size after combination therapy was much smaller than that after the other therapies (Figure 7B). This result indicated that combination therapy could effectively inhibit tumor growth and that the antitumor effect of combination therapy was better than that each therapy separately. The curves for the mouse body weight relative to time after different treatments are shown in Figure 7D. The body weights of the mice in the five groups were measured during the treatments, and no significant weight loss was discovered, implying the good biosafety of our treatments.

**Figure 8:**
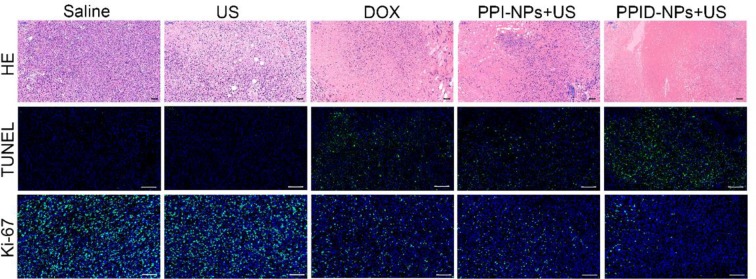
H&E staining and TUNEL and Ki-67 assays of tumor tissue slices after various treatments (scale bar, 50 μm).

Tissue histological analysis, including H&E staining and the TUNEL and Ki-67 assays was carried out to confirm the therapeutic effect (Figure 8). According to the H&E staining and TUNEL assay results, almost none of the tumor cells were dead in the saline or US-only group, suggesting that US has no obvious antitumor activity. In contrast, the DOX and PPI-NP + US groups showed considerable cell death in the H&E staining and TUNEL assay result, indicating that either chemotherapy or SDT by itself has a significant antitumor effect. However, the PPID-NP combined with US irradiation group exhibited better antitumor efficacy than each of the single therapy groups. The H&E staining and TUNEL assay results confirmed that most of the tumor cells were destroyed. For the Ki-67 assay, the saline and US-only group showed many proliferative cells stained with green fluorescence. By contrast, fewer proliferative cells were observed in the other three groups. Compared with the DOX or PPI-NP + US group, the PPID-NP + US group had weaker green fluorescence intensity, indicating that most tumor cells had reduced proliferation and decreased malignancy. These pathology assays showed that PPID-NP with US treatment was more effective than treatment with PPI-NPs + US or DOX alone, suggesting that synergistic chemotherapy and sonodynamic therapy could provide greatly enhanced antitumor therapeutic efficacy.

### Pharmacokinetics and Biodistribution

**Figure 9:**
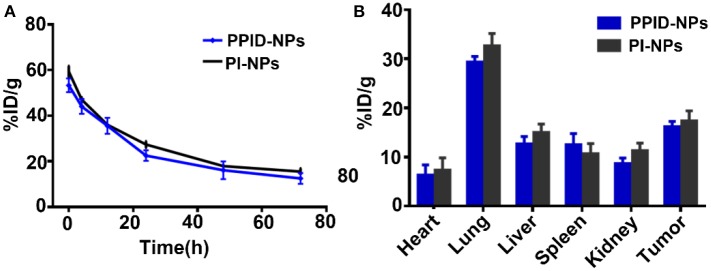
**(A)** *in vivo* pharmacokinetic curves over a span of 72 h after intravenous injection of PPID-NPs or PI-NPs. **(B)** Biodistribution of PPID-NPs or PI-NPs at 24 h after the injection.

To investigate the *in vivo* metabolism of the NPs, tumor-bearing BALB/c mice were injected with 200 μL of PPID-NPs or PI-NPs (the concentration of IR780 was 2.0 mg/kg) through the tail vein. At different time points (0, 4, 12, 24, 48, and 72 h), submandibular vein blood of the mice was collected for UV-Vis-NIR spectrophotometry analysis. As shown in Figure 9A, after the first 24 h, the concentration of PPID-NPs in blood was decreased to 35.6% of the injected dose per gram of tissue (ID/g), and after the 3th 24 h, the concentration of PPID-NPs in blood was down to 12.5% ID/g. Similarly, the concentration of PI-NPs in blood was 36.0% ID/g in the first 24 h and 15.5% ID/g in the 3th 24 h, there was no significantly difference between two groups at each tested time point.

For the *in vivo* biodistribution analysis of the NPs, the major organs and tumors of the mice were collected, lysed in DMSO and homogenized to test the IR780 contents with a UV-Vis-NIR spectrophotometer. After injection of NPs for 24 h, three mice in each group were euthanized to measure the NPs distribution in the major organs. As shown in Figure 9B, the majority of the NPs accumulated in the lung due to the enriched RES uptake, the concentration of PPID-NPs and PI-NPs in lungs were 29.3 and 32.7%, respectively, which has no significant different between two groups. The concentration of PPID-NPs and PI-NPs in tumors were 16.2 and 17.3%, respectively, which was higher than other RES organs, such liver, spleen. That maybe contribute to the mitochondria-targeted ability of IR780, which were reported by other published articles (Zhang et al., 2018, 2019; Yang et al., 2020).

## Conclusion

In summary, we successfully developed an oxygen-sufficient nanoplatform, PPID-NPs, to enhance the efficiency of synergistic sonodynamic-chemotherapy in hypoxic tumors. On the one hand, PPID-NPs can deliver oxygen to tumors and enhance the efficacy of oxygen-dependent SDT. On the other hand, PPID-NPs used as a drug delivery system could accelerate DOX release under US exposure. Importantly, the *in vitro* and *in vivo* results confirmed that chemo-sonodynamic therapy possesses high efficiency for anticancer therapy. Therefore, PPID-NPs, as an oxygen-sufficient nanoplatform, could be used as an ideal therapeutic system for hypoxic tumors.

## Data Availability Statement

All datasets generated for this study are included in the article/supplementary material.

## Ethics Statement

The animal study was reviewed and approved by Ethics Committee of Central South University.

## Author Contributions

BH and CN: conceptualization and data curation. BH and LW: methodology. SC, WP, YX, and ZJ: analysis and investigation. BH: writing original draft preparation. CN: writing-review and editing. LW: supervision.

## Conflict of Interest

The authors declare that the research was conducted in the absence of any commercial or financial relationships that could be construed as a potential conflict of interest.
